# Human amniotic epithelial cells alleviate ischemia-reperfusion injury of steatotic livers through mediating PAK1/AMPK-dependent autophagy

**DOI:** 10.1016/j.gendis.2024.101343

**Published:** 2024-05-29

**Authors:** Xun Qiu, Hanzhi Xu, Yawen Tan, Jinying Li, Zhoucheng Wang, Zhengxing Lian, Xuyong Wei, Luyang Yu, Kai Wang, Xiao Xu

**Affiliations:** a Zhejiang University School of Medicine, Hangzhou, Zhejiang 310058, China; b Key Laboratory of Integrated Oncology and Intelligent Medicine of Zhejiang Province, Hangzhou, Zhejiang 310006, China; c Institute of Genetics and Regenerative Biology, College of Life Sciences, Hangzhou, Zhejiang 310058, China; d Center for Stem Cell and Regenerative Medicine, Zhejiang University, Hangzhou, Zhejiang 310058, China; e Department of Hepatobiliary & Pancreatic Surgery and Minimally Invasive Surgery, Zhejiang Provincial People's Hospital (Affiliated People's Hospital), Hangzhou Medical College, Hangzhou, Zhejiang 310014, China; f School of Clinical Medicine, Hangzhou Medical College, Hangzhou, Zhejiang 310059, China; g Institute of Translational Medicine, Zhejiang University, Hangzhou, Zhejiang 310000, China

Hepatic steatosis is prevalent worldwide and is characterized as excessive lipid accumulation with/without inflammation and injury in the liver. Hepatic ischemia-reperfusion (HIR) injury commonly occurs in the process of hemorrhagic shock, liver surgery, and liver transplantation, and impairs liver function by inhibiting the electron transport chain in mitochondria during ischemia stage and producing large amount of reactive oxygen species (ROS) during reperfusion stage.^1^ Steatotic livers are more susceptible to HIR injury due to redundant lipid ROS and immune imbalance, which could induce dysregulation of autophagy.^2^ Therefore, it is of great importance to explore viable strategies for the protection of steatotic livers from HIR injury. Human amniotic epithelial cells (hAECs), which are regarded as a promising cell type for cell-based therapies due to low immunogenicity and tumorigenicity, stem-cell-like plasticity, and paracrine properties, have been discovered to act in anti-inflammation and function repair of multiple tissues and organs (*e.g.*, skin, liver, kidney, and lung).^3^ However, the role of hAECs in steatotic liver HIR injury has not been reported. Here, our results showed that hAECs could ameliorate HIR injury of steatotic livers through modulating p21-activated kinase 1 (PAK1)/AMP-activated protein kinase (AMPK)-dependent autophagy.

Six-week-old mice were fed an eight-week 60% Kcal high-fat diet, followed by hepatic ischemia and reperfusion surgery. All mice were divided into three groups (Sham, HIR, and HIR with hAECs treatment groups) and the modulatory effects of hAECs on HIR injury of steatotic livers were assessed. Results showed that liver function (ALT and AST) (Fig. 1A, B) and hepatic levels of lipid peroxidation (Fig. 1C) were remarkably improved in the hAECs-treated group. The hAECs also significantly alleviated hepatic apoptosis and necrosis, evidenced by down-regulated levels of Bax and cleaved-caspase3 and decreased necrotic areas (Fig. 1D, E). These results showed that hAECs could improve HIR injury of steatotic livers.

**Figure 1:**
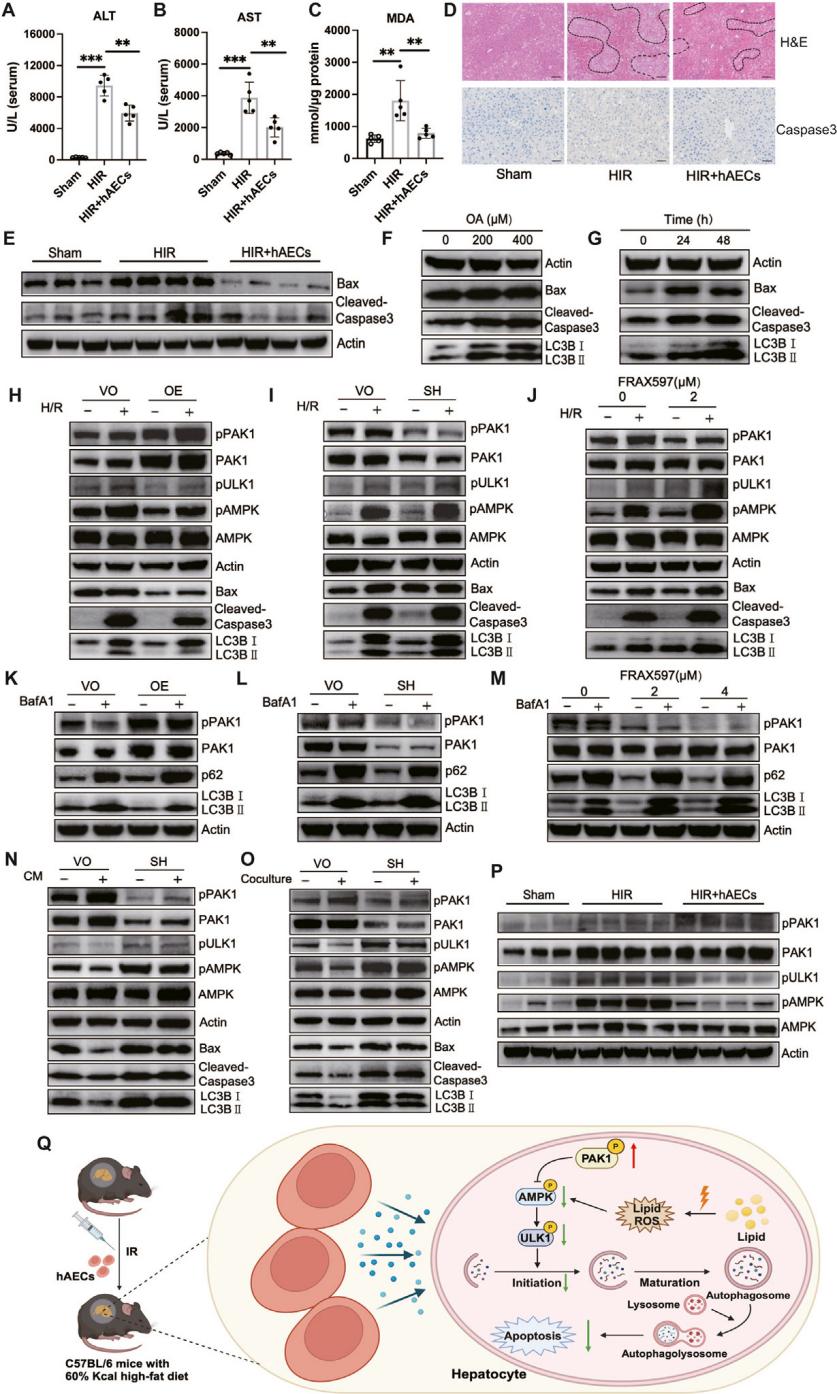
Human amniotic epithelial cells alleviate ischemia-reperfusion injury of steatotic livers through mediating PAK1/AMPK-dependent autophagy. Mice fed with an eight-week high-fat diet were subjected to liver partial warm ischemia for 90 min followed by 6 h of reperfusion. **(A**–**C)** The serum levels of ALT (A) and AST (B) and the hepatic level of MDA (C) were measured with microplate methods. **(D)** Histological slices of liver tissues were stained with Caspase3 and hematoxylin/eosin. **(E)** Western blotting analysis of apoptosis-related proteins in liver tissues. **(F, G)** Western blotting analysis of AML12 cells treated with different concentrations of oleic acid for 48 h (F), or treated with 400 μM oleic acid for 0, 24, or 48 h (G). **(H, I)** Steatotic hepatocytes with different PAK1 expression were treated with/without hypoxia-reoxygenation (H/R), then the levels of proteins were determined by western blotting. **(K**–**M)** Steatotic hepatocytes with different PAK1 expression were treated with/without bafilomycin A1 and then the levels of proteins were determined by western blotting. **(N, O)** Steatotic hepatocytes with different PAK1 expression were subjected to H/R and treated with/without conditioned media from hAECs (CM) (N) or cocultured with/without hAECs (O) during reoxygenation, then the levels of proteins were determined by western blotting. **(P)** Western blotting analysis of PAK1, pPAK1, and autophagy-related proteins in liver tissues. **(Q)** The diagram of experimental results. The hAECs could alleviate HIR injury of steatotic livers by promoting the activation of PAK1 and then mitigating the AMPK/ULK1-dependent autophagy in hepatocytes. The figure was generated with BioRender. The black dotted line indicated a necrotic area. Data were represented as mean ± standard deviation. *n* = 5/group. ∗∗*P* < 0.01, ∗∗∗*P* < 0.001. Scale bar, 50 μm.

The *in vitro* model of steatotic hepatocytes was constructed by treating normal hepatocytes with 400 μM oleic acid for 48 h. Compared with normal hepatocytes, steatotic hepatocytes were more sensitive to hypoxia-reoxygenation (H/R) injury and exhibited higher levels of autophagy (Fig. S1; Fig. 1F, G). PAKs are serine–threonine kinases and play critical roles in various biological processes.^4^ PAK4 has been reported to be up-regulated in HIR and its inhibition could alleviate inflammation and necrosis of hepatocytes through up-regulating the transcriptional activity of nuclear factor erythroid 2-related factor 2 (Nrf2).^5^ PAK1 levels have been indicated to be increased in both steatotic and non-steatotic livers after transplantation,^4^ while its role and molecular mechanisms in HIR remain elusive. Therefore, we explored the effects of PAK1 in H/R injury and its association with autophagy. As shown in Figure 1H, overexpression of PAK1 suppressed AMPK/Unc-51-like autophagy activating kinase 1 (ULK1)-dependent autophagy, thus alleviating H/R injury. Consistently, hepatocytes with PAK1 inhibition showed enhanced activation of the AMPK/ULK1 pathway and increased levels of apoptosis (Fig. 1I, J). To further investigate the underlying mechanisms that PAK1 suppressed autophagy, steatotic hepatocytes with different PAK1 expression were treated with or without bafilomycin A1. As shown in Figure 1K, PAK1-overexpressing hepatocytes with or without bafilomycin A1 treatment exhibited decreased LC3B II expression and increased p62 expression, implying that PAK1 regulated the initiation of autophagy rather than the fusion of autophagosome and lysosome. Consistently, chemical or genetic inhibition of PAK1 facilitated the initiation of autophagy (Fig. 1L, M). These findings indicated that PAK1 suppressed the H/R injury of steatotic hepatocytes by inhibiting AMPK/ULK1-dependent autophagy initiation.

Next, we evaluated whether PAK1 was necessary for hAECs to alleviate HIR injury of steatotic livers. As shown in Figure 1N and O, H/R injury was improved when treated with conditioned media from hAECs (CM) or cocultured with hAECs, and PAK1 inhibition in hepatocytes impaired the anti-autophagy and anti-apoptosis effects of hAECs. In addition, the activated form of PAK1 (pPAK1) was up-regulated in the hAECs-treated group, while levels of autophagy-associated biomarkers including pAMPK, pULK1, and LC3B II were down-regulated (Fig. 1P), verifying the *in vitro* results that hAECs alleviated HIR injury of steatotic livers through modulating PAK1-associated autophagy.

In conclusion, steatotic livers were more sensitive to HIR injury and exhibited higher levels of autophagy, while hAECs could improve HIR injury by increasing the activation of PAK1 and thus mitigating the AMPK/ULK1-dependent autophagy in steatotic hepatocytes (Fig. 1Q). Our findings first demonstrated the protective effects of hAECs on HIR injury of steatotic livers, suggesting that hAECs would be a potential therapeutic approach to attenuate HIR injury of steatotic livers in the settings of major hepatectomy and transplantation.

## Ethics declaration

All mice were obtained from the Zhejiang Center of Laboratory Animals (Zhejiang, China). The experimental protocols were authorized by the Institutional Review Board of Institutional Animal Care and Use Committee, Zhejiang Center of Laboratory Animals (No. ZJCLA-IACUC-20010152).

## Author contributions

X.Q., H.X., and K.W. designed the research. X.Q., H.X., Y.T., J.L., X.W., K.W., and X.X. wrote and revised the manuscript. X.Q., H.X., Y.T., J.L., Z.W., and Z.L. performed the experiments. K.W. and X.X. contributed to the project administration and funding acquisition. All authors read and approved the final manuscript.

## Conflict of interests

The authors declared no competing interests.

## Funding

This work was supported by the 10.13039/501100012166National Key Research and Development Program of China (No. 2021YFA1100503), 10.13039/501100004731Zhejiang Provincial Natural Science Foundation (China) (No. LY22H160048), and Key Research & Development Program of Zhejiang Province (China) (No. 2022C03108).
