# Supplementary material for: Human amniotic epithelial cells alleviate ischemia-reperfusion injury of steatotic livers through mediating PAK1/AMPK-dependent autophagy
Source: Genes Dis. 2024 May 29;12(2):101343. doi: 10.1016/j.gendis.2024.101343 (PMC11625314; doi:10.1016/j.gendis.2024.101343)
Supplement: Multimedia component 1 [file mmc1.docx]

**Reagents**

PAKs inhibitor FRAX597 (S7271) and Bafilomycin A1 (BafA1) (S1413) were purchased from Selleck (TX, USA). Oleic acid (OA) (Sigma-Aldrich, MO, USA) was dissolved in 10% BSA with a stock concentration of 10 mM.

**Cell culture**

AML12 cell line was purchased from Shanghai Institute of Cell Biology (China) and cultured at 37℃ in an atmosphere containing 5% CO_2_ in high glucose DMEM (Gibco, NY, USA) supplemented with 10% fetal bovine serum (Wisent, Montreal, Canada). HAECs were purchased from iCELL biotechnology (Shanghai, China) and cultured at 37℃ in an atmosphere containing 5% CO_2_ in DMEM (Gibco, NY, USA) supplemented with 10% fetal bovine serum (Wisent, Montreal, Canada), 1% non-essential amino acid (Gibco, NY, USA), 1% antibiotic-antimycotic (Gibco, NY, USA), 2 mM L-glutamine (Solarbio, Beijing, China), 1 mM sodium pyruvate (Gibco, NY, USA), 55 μM 2-mercaptoethanol (Gibco, NY, USA) and 10 ng/mL human EGF (Peprotech, NJ, USA). Approximately 2×10^6^ hAECs were cultured in a 100-mm dish up to 90% confluency, and the basic media was replaced with 10 ml serum- and glucose-free DMEM for 24 hours, which was centrifuged at 2,000 rpm for 10 min at 4℃ to remove cell debris and was 10-fold concentrated using Amicon Ultra-15 ultrafiltration conical tubes with membranes selective for 3 kDa (Millipore, MA, USA) to generate conditioned media from hAECs (CM).

**Generating AML12 cells with PAK1 overexpression or knockdown**

Cells were plated in a six-well plate at 30% confluent cultures and transfected with lentiviral particles containing PAK1 plasmid DNA, specific short hairpin RNA (shRNA) or the corresponding empty vector (Genechem, Shanghai, China). Then, cells were selected by 2 μg/mL puromycin (Selleck, TX, USA) and collected for gene expression assays.

**Oil red O staining**

For evaluating the lipid accumulation, cells were fixed in paraformaldehyde for 15 min, followed by washing with PBS. Then, cells were soaked in 60% isopropanol for 5 min and then stained with Oil red O (Sigma-Aldrich, MO, USA) at room temperature for 30 min. Finally, cells were counterstained with haematoxylin for 30 s and images were taken by Olympus digital camera.

**Steatotic hepatocytes** **hypoxia-****reoxygenation (H/R) model**

Cells that have been treated with 400 μM oleic acid for 48 hours, were cultured with glucose-free DMEM at 37°C for 24 hours in a humidity airtight chamber equilibrated with 95% N_2_-5% CO_2_. The medium was then replaced with high glucose DMEM supplemented with 10% fetal bovine serum, and cells were further incubated at 37°C in a 95% O_2_-5% CO_2_ atmosphere for 12 hours.

**Coculture experiments**

Steatotic hepatocytes (5×10^5^) in a six-well plate were washed by PBS after hypoxia, and then cocultured with/without hAECs (coculture ratio 1:1) during reoxygenation. HAECs were seeded on the upper chamber of the Transwell filter system (pore size of 0.4 μm; Corning, NY, USA).

**Animal experiments**

Six weeks-old female C57BL/6 mice were fed 60% Kcal high-fat diet (HFD) for 8 weeks, followed by hepatic ischemia and reperfusion surgery. Hepatic ischemia was established by occluding the blood flow to the left and medial lobes of liver (portal vein and the hepatic artery) for 90 min. The clamp was removed and followed by reperfusion. In the HIR group, 10^6^ hAECs or equivalent amount of PBS was injected through the portal vein during reperfusion. Sham control mice were performed the same procedures without vessel occlusion. After 6 hours, mice were euthanized, and blood samples and liver tissues were collected.

**Western blotting analysis**

Cells and mouse liver tissues were lysed with RIPA buffer (FUDE, Hangzhou, China) containing phosphatase inhibitors and protease inhibitors. The cell lysates were subjected to SDS-PAGE and blotted wetly onto a PVDF membrane. The membranes were blocked in 5% non-fat milk and then incubated with the primary antibody overnight at 4 °C followed by incubation with the secondary antibody for 1-2 hours at room temperature. The protein bands were visualized by chemiluminescence. The primary antibodies included rabbit anti-β-actin (Abclonal, Wuhan, China), rabbit anti-PAK1 (CST, MA, USA), rabbit anti-pPAK1 (CST, MA, USA), rabbit anti-pAMPKα (Thr172) (CST, MA, USA), rabbit anti-AMPKα (CST, MA, USA), rabbit anti-pULK1 (Ser555) (ABclonal, Wuhan, China), rabbit anti-p62 (Peprotech, NJ, USA), rabbit anti-LC3B (Abways, Beijing, China), rabbit anti-Bax (CST, MA, USA), and rabbit anti-Cleaved caspase3 (CST, MA, USA).

**MDA and liver function (ALT/AST) assays**

According to the manufacturer's instructions, the Lipid Peroxidation MDA Assay Kit (Beyotime Biotechnology, Shanghai, China), ALT Assay Kit (Jiancheng Bio, Nanjing, China), and AST Assay Kit (Jiancheng Bio, Nanjing, China) were used to detect the hepatic lipid peroxidation level and liver function.

**Immunohistochemistry and histology analysis**

The liver tissues isolated from the mice were fixed with buffered 4% paraformaldehyde overnight and paraffin‐embedded (Biosharp, Heifei, China). Immunohistochemistry (IHC) for Caspase3 and Hematoxylin and Eosin (H&E) staining were performed according to the manufacturer's protocols.

**Statistical analysis**

Statistical analysis was performed using GraphPad Prism (version 8.0.1) and R language (version 4.0.3). Comparison of groups was achieved with Student’s t-test. Values of p<0.05 were considered as significantly different.
