# Supplementary material for: Human amniotic epithelial cells alleviate ischemia-reperfusion injury of steatotic livers through mediating PAK1/AMPK-dependent autophagy
Source: Genes Dis. 2024 May 29;12(2):101343. doi: 10.1016/j.gendis.2024.101343 (PMC11625314; doi:10.1016/j.gendis.2024.101343)
Supplement: Multimedia component 2 [file mmc2.docx]

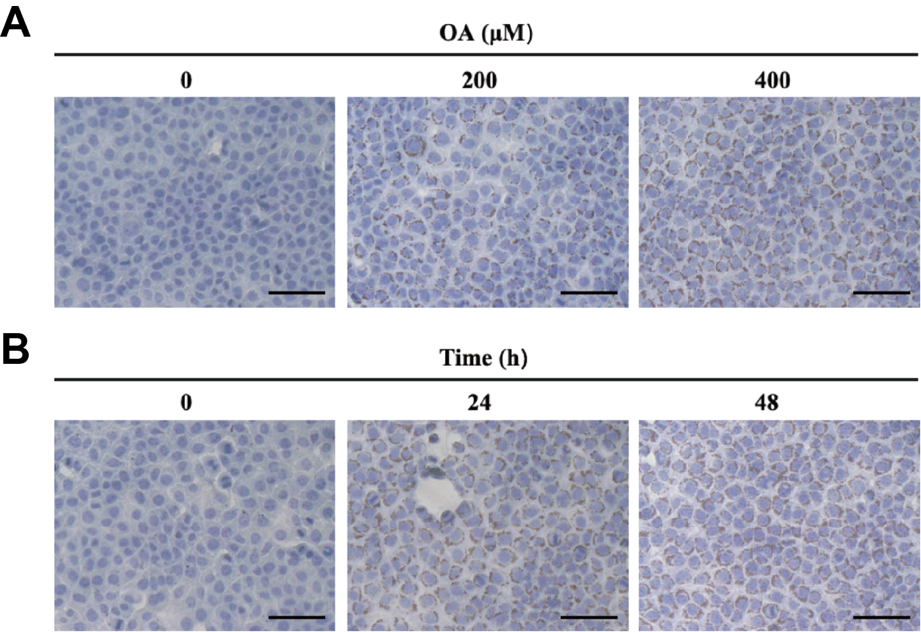


**Figure S1** Steatosis led to higher levels of apoptosis and autophagy in hepatocytes after H/R. Oil red O staining of AML12 cells that were treated with different concentrations of oleic acid for 48 hours **(A)**, or treated with 400 μM oleic acid for 0, 24 or 48 hours **(B)**. Scale bar, 25 μm.
